# Anesthetic Management With Peripheral Nerve Blocks and Sedation for Popliteal Artery Aneurysm Repair in a Patient With Severe Pulmonary Dysfunction: A Case Report

**DOI:** 10.7759/cureus.79933

**Published:** 2025-03-02

**Authors:** Toshiki Yoshioka, Kazuyoshi Ishida, Hiromasa Irie

**Affiliations:** 1 Department of Anesthesiology, Kurashiki Central Hospital, Kurashiki, JPN

**Keywords:** peripheral nerve block, popliteal artery aneurysm, postoperative pulmonary complications, regional anesthesia, severe pulmonary dysfunction

## Abstract

Postoperative pulmonary complications (PPCs), such as pneumonia, atelectasis, and respiratory failure, pose a significant challenge, particularly in patients with compromised respiratory function. Regional anesthesia is well-known for its numerous advantages, including its effectiveness in mitigating PPCs by circumventing the need for general anesthesia and intubation.

A 79-year-old man with no significant medical history other than chronic obstructive pulmonary disease presented with left lower limb swelling, gait disturbance, and cold sensation. Computed tomography revealed a 5 cm left popliteal artery aneurysm, which was diagnosed as the cause of the patient’s symptoms. He underwent popliteal artery aneurysm repair using peripheral nerve blocks (PNBs) and sedation. Sciatic, femoral, and obturator nerve blocks with 0.25% levobupivacaine provided effective analgesia. Despite intraoperative challenges, such as elevated partial pressure of carbon dioxide (PaCO_2_) and prolonged operative time due to vascular reconstruction revisions, the patient remained hemodynamically stable throughout the procedure, and PNBs allowed the preservation of spontaneous respiration. The patient was discharged on postoperative day 15 without pneumonia or respiratory failure. This case underscores the utility of PNBs and sedation in mitigating PPCs like pneumonia.

## Introduction

Postoperative pulmonary complications (PPCs), including pneumonia, atelectasis, and respiratory failure, are a significant concern in patients with impaired respiratory function, leading to increased morbidity and mortality [1,2]. To mitigate these risks, regional anesthesia is often preferred over general anesthesia, as it eliminates the need for intubation and may improve perioperative outcomes [3,4]. In high-risk patients, a combination of regional anesthesia and sedation has been successfully used for lower extremity surgeries, reducing respiratory complications [5,6].

This report describes a 79-year-old man with no significant medical history other than chronic obstructive pulmonary disease (COPD) who underwent repair of a popliteal artery aneurysm under regional anesthesia and sedation. Although laboratory investigations revealed only mild anemia, pulmonary function tests indicated severe obstructive ventilatory impairment, with a forced expiratory volume in one second (FEV_1_) of 1,260 mL. Additionally, the patient required home oxygen therapy at 3 L/min during exertion. Due to the status and the strong concern for PPCs, we planned a combination of peripheral nerve blocks (PNBs) and sedation. Despite an extended operative duration due to multiple vascular reconstruction revisions, the procedure was successfully completed under regional anesthesia and sedation, and the patient was discharged without PPCs.

This case was presented at the 10th Annual Meeting of the Japanese Society of Regional Anesthesia in 2023.

## Case presentation

The patient was a 79-year-old man who presented with swelling, coldness, and paresthesia in the left lower extremity. He had a history of COPD and a cumulative smoking history of 122 pack-years, having smoked 40 cigarettes per day from the age of 17 to 78. Regarding respiratory function assessment, the six-minute walk test revealed a decline in oxygen saturation (SpO_2_)​​​​​ from 96% to 84% within three minutes, necessitating termination of the test. Consequently, home oxygen therapy (3 L/min during exertion) was initiated. The patient was 166 cm tall and weighed 64 kg. Auscultation revealed mild wheezing without cardiac murmurs. Additional physical findings included left lower extremity swelling and mild paresthesia. There was no significant past medical history, family history, or psychiatric history. Computed tomography identified a 5 cm left popliteal artery aneurysm, for which surgical repair was scheduled. Perioperative blood tests demonstrated a hemoglobin level of 13.3 g/dL, consistent with mild anemia. No other abnormalities were noted in biochemical, complete blood count, or coagulation parameters. Preoperative arterial blood gas analysis revealed a partial pressure of carbon dioxide (PaCO_2_) of 44 mmHg. Pulmonary function tests showed a total lung capacity of 3,210 mL, an FEV₁ of 1,260 mL (39% of the predicted value), and a peak expiratory flow of 5.2 L/sec (67% of the predicted value). Transthoracic echocardiography did not reveal any significant abnormalities. The Assess Respiratory Risk in Surgical Patients in Catalonia (ARISCAT) score was 34 points, categorizing him as being at moderate risk for PPCs [7]. Considering the risk of PPCs, we adopted a combination of PNBs and sedation. Continuous monitoring, including three-lead electrocardiogram, oxygen saturation, invasive arterial pressure measurement using an arterial catheter, bispectral index monitor, and end-tidal CO_2_, was implemented.

**Figure 1:**
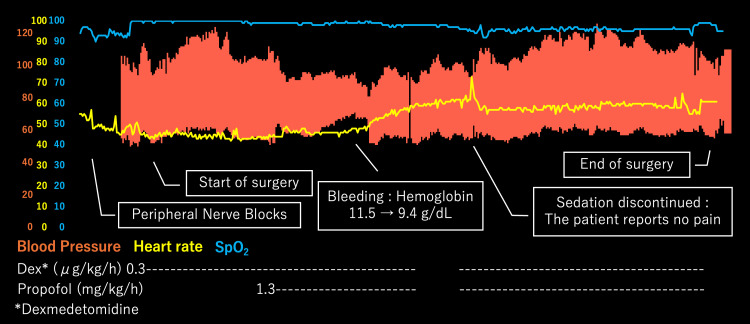
The anesthesia record Sedation was interrupted, and the patient was awakened, but no pain complaints were reported.

Initially, in the non-sedated state, an ultrasound-guided sciatic nerve block was performed via the subgluteal approach in the lateral decubitus position using 20 mL of 0.25% levobupivacaine. Femoral and obturator nerve blocks were subsequently administered in the supine position with 20 mL and 10 mL of 0.25% levobupivacaine, respectively. During the administration of the PNBs, a local anesthetic was injected prior to needle insertion. The surgery was performed with the patient in the supine position under sedation with propofol (1.3 mg/kg/h) and dexmedetomidine (0.3 μg/kg/h). Oxygen was supplied at 3 to 5 L/min via a face mask. Intraoperatively, arterial blood gas analysis was conducted as needed. At anesthesia induction, PaCO_2_ was 47 mmHg, increasing slightly to 49 mmHg while in the supine position. However, after transitioning to the prone position during the middle phase of the procedure, PaCO_2_ increased to 64 mmHg, likely due to suboptimal head and neck positioning. After the sedation was temporarily interrupted and the patient briefly awakened to adjust the head and neck position, PaCO_2_ improved to 49 mmHg. At that time, he reported only mild numbness in the left lower extremity and no pain. Intraoperatively, hemoglobin levels decreased from 11.5 g/dL to 9.4 g/dL due to blood loss, prompting the transfusion of 500 mL of salvaged blood. The total operative time was 6 hours and 10 minutes, extended due to additional reconstruction for a popliteal artery dissection. There was no need for conversion to general anesthesia or additional local anesthetic administration. The total fluid volume administered was 2,850 mL, with a urine output of 565 mL and an estimated blood loss of 1,111 mL (refer to Figure 1 for fluctuations in vital signs).

Postoperatively, the patient was admitted to the high dependency unit. His respiratory status remained stable, with PaCO_2_ maintained between 38 and 43 mmHg. The next day, he was transferred to the general ward. His postoperative course was uneventful, and he was discharged on postoperative day 15.

## Discussion

In this case, a popliteal artery reconstruction was planned with the great saphenous vein as a graft, necessitating analgesic coverage from the medial thigh to the popliteal fossa. Femoral, sciatic, and obturator nerve blocks were performed based on a previous report describing the efficacy of PNBs for popliteal artery aneurysms [8]. A sciatic nerve block distal to the subgluteal area was deemed insufficient to achieve the required lower extremity anesthesia in this case; therefore, a subgluteal approach was chosen. The parasacral approach was not used because most sciatic nerve blocks at our institution are routinely performed via the subgluteal approach, a technique with which we are highly experienced. According to the above report [8], the average duration of the popliteal artery repair procedures was approximately four hours. In this case, the procedure exceeded four fours, but the PNBs provided adequate analgesia. A sciatic nerve block with 0.5% levobupivacaine typically provides adequate analgesia for 10 to 16 hours [9,10]. As demonstrated in this case, with an appropriate technique, adequate analgesia can be achieved with 0.25% levobupivacaine for prolonged surgery. Sedation was interrupted midway, and direct communication was established with the patient. At that time, the patient reported feeling no pain, confirming the effectiveness of PNBs.

For patients at elevated respiratory risk, anesthetic management should focus on mitigating the risk of PPCs. The ARISCAT score, a widely accepted risk assessment tool for PPCs, placed this patient in the moderate risk category with a preoperative score of 34 points. Preventing PPCs necessitates a multifaceted approach, including the correction of anemia, intraoperative lung-protective ventilation strategies, careful management of neuromuscular blockade, and avoidance of positive-pressure ventilation [1,11,12]. Recent studies indicate that PPCs can be reduced after lower extremity surgery by preserving respiratory function and by employing regional anesthesia, particularly neuraxial anesthesia [13-15]. PNBs, akin to neuraxial anesthesia, circumvent the need for general anesthesia and the associated use of neuromuscular blockade and endotracheal intubation. Additionally, PNBs offer specific advantages over neuraxial anesthesia, particularly in cases requiring intraoperative heparinization or for prolonged surgery, as in this case.

The benefits of regional anesthesia for lower extremity surgery in high-risk cardiovascular patients have recently been highlighted in terms of reduced postoperative morbidity and mortality. In patients undergoing lower limb amputation, regional anesthesia has been shown to reduce the incidence of postoperative respiratory failure and the need for blood transfusion [16,17], and, particularly in patients with chronic heart failure, regional anesthesia is associated with reduced mortality [18]. Previous studies have shown that in patients undergoing bypass surgery, regional anesthesia reduces delirium, fluid administration, and catecholamine use [19] but does not affect mortality or length of hospital stay [20]. However, a recent large cohort study reported reductions in 30-day mortality and postoperative complications in patients with cardiovascular disease [6], suggesting the effectiveness of regional anesthesia in high-risk patients. Although the patient in this case did not have cardiovascular disease, these findings underscore the importance of anesthesiologists being proficient in regional anesthesia to optimize care.

## Conclusions

This case highlights the advantages of PNBs combined with sedation in patients at high risk for PPCs. By preserving spontaneous respiration and avoiding general anesthesia, this anesthetic approach successfully mitigated respiratory risks in a patient with severe pulmonary dysfunction undergoing popliteal artery aneurysm repair. Despite intraoperative challenges, effective regional anesthesia management allowed for a stable perioperative course without the need for conversion to general anesthesia. This case underscores the importance of regional anesthesia as a valuable option for managing patients with significant pulmonary comorbidities.
